# Freshwater lake ecosystem shift caused by social-economic transitions in Yangtze River Basin over the past century

**DOI:** 10.1038/s41598-018-35482-5

**Published:** 2018-11-21

**Authors:** Ke Zhang, Xiangdong Yang, Giri Kattel, Qi Lin, Ji Shen

**Affiliations:** 1 State Key Laboratory of Lake Science and Environment, Nanjing Institute of Geography and Limnology, Chinese Academy of Sciences, Nanjing, 210008 China; 2 Environmental Hydrology and Water Resources Group, Department of Infrastructure Engineering, The University of Melbourne, Parkville, Victoria 3010 Australia

## Abstract

Global lake systems have undergone rapid degradation over the past century. Scientists and managers are struggling to manage the highly degraded lake systems to cope with escalating anthropogenic pressures. Improved knowledge of how lakes and social systems co-evolved up to the present is vital for understanding, modeling, and anticipating the current and future ecological status of lakes. Here, by integrating paleoenvironmental, instrumental and historical documentary resources at multi-decadal scales, we demonstrate how a typical shallow lake system evolved over the last century in the Yangtze River Basin, an urbanized region containing thousands of shallow lakes. We find abrupt ecological shift happened in the lake ecosystem around the 1970s, with the significant reorganization of macrophyte, diatom and cladocera communities. The lake social-ecological system went through three stages as the local society transformed from a traditional agricultural before 1950s to an urbanized and industrialized society during the recent thirty years. The timing and interaction between social, economic and ecological feedbacks govern the transient and long-term dynamics of the freshwater ecosystem. Our results highlight the importance of accounting for the long-term dynamics and feedbacks between ecological, social and economic changes when defining safe operating spaces for sustainable freshwater ecosystem management.

## Introduction

Freshwater lake ecosystems are among the most valuable and heavily used natural systems worldwide, and they provide important ecosystem services to many millions of people^1,2^. Widespread decline in ecological conditions, loss of biodiversity and dramatic shift in ecosystem structure and function have been reported from different parts of the world^3–5^. Scientists and managers face many challenges to manage heavily degraded lake ecosystems. For instance, the effects of natural perturbations and human activities in modern landscapes may be accentuated by events in previous decades or even centuries^6^, and the historical legacy makes it difficult to fully understand the mechanism of current degradation^7^. In some cases, restoration and bringing the ecosystem back to the “references condition” may not be practical as many ecosystems have been irreversibly altered into “novel ecosystems”^8,9^. Further, current socioeconomic constraints related to food production and the livelihood of people within the lake catchment can ultimately complicate the full restoration of freshwater lake ecosystems^10,11^.

A deeper understanding of how social and ecological system co-evolved through time is important for modeling, understanding, and anticipating current and future social-ecological system^12,13^. Long-term perspective can help to observe the nature of legacies and contingencies: such as the slow and fast process^14^, the existence of threshold^15^, and the convergence and divergence of system and variable trajectories^16,17^. These system behaviors can give crucial insight into the functioning of contemporary social-ecological systems^18^. New frameworks and models are emerging for investigating the interaction between long periods of stability and abrupt change in social-ecological systems^19,20^. For instance, Cumming *et al*. provided a conceptual framework to illustrate how society over-exploit ecosystems through time and leading to “green (sustainable)” or “red (unsustainable)” loops^21^. However, far fewer empirical studies attempt to combine ecological, social and economic perspectives to inform about coupled social-ecological change at multi-decadal scales^22^. The lack of long-term biophysical records is a major barrier, as direct observations from monitoring and survey were usually too short or too limited in scope to provide a comprehensive record^23^. Palaeoenvironmental sciences could provide continuous multi-decadal records for an array of ecosystem states, process and services^13,18,24^. Although there are increasing paleoecological studies examining the effects of land use change on lake ecosystems, fewer studies address these complex interactions from a social-ecological perspective^25–27^.

**Figure 1:**
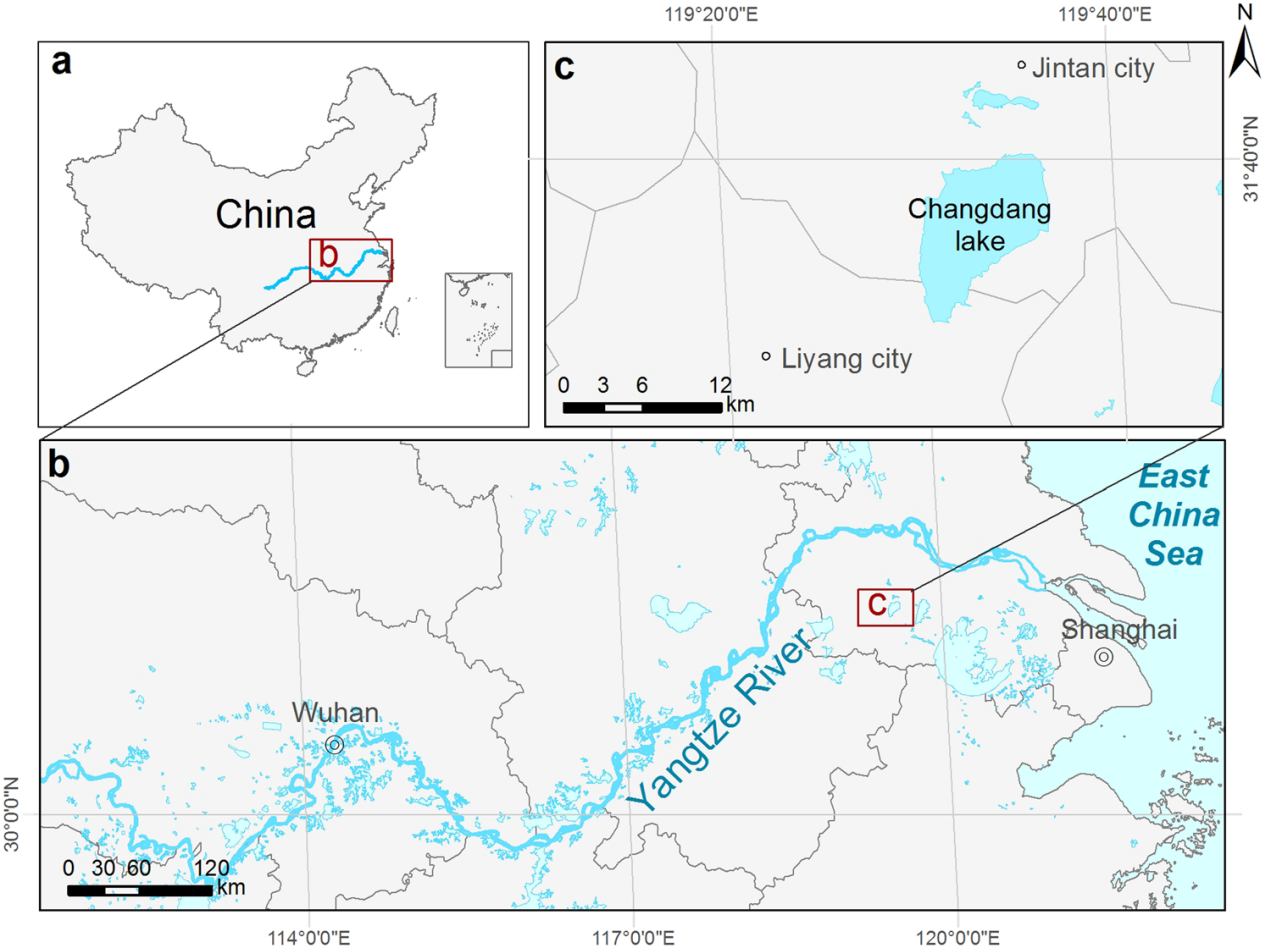
Location of the study site, Changdang Lake (**c**) in the middle and lower Yangtze River Basin (**b**), China (**a**). Waterbody colored in light blue in (**b**) shows hundreds of lakes located in this region along the Yangtze River.

In this study, we take a historical evolutionary approach to generate new insights into the lake ecosystem and social change within the past century, using the Changdang Lake from the Lower Yangtze River Basin as a case study (Fig. 1). The lakes along the low Yangtze River are one of the largest groups of shallow lakes in the world^28^, and this region experienced dramatic social and ecological transition during the last centuries, which exemplifies the changes that have occurred across lake ecosystems globally. More than 80% of lakes examined in this region are seriously degraded and eutrophied^29^. The government has made great efforts to restore the lake’s ecosystem, with little progress so far^30^. Previous attempts have failed because many of them have focused only on the most recent symptoms of the problems rather than on their deep historical cause^31^. Many contemporary studies often seek to explain current conditions by the most recent events only^32,33^. A key challenging facing lake managers in China and elsewhere is to develop a deeper, holistic understanding of lake ecosystem dynamics through time.

Here, we integrate paleoenvironmental, instrumental and historical documentary records to examine multi-decadal changes of social-ecological system in Changdang Lake, with two key objectives; (1) Reveal the trajectories of change that have produced the current situation of the lake ecological system; (2) How the interactions, feedback between lake social and ecological systems changed during the last century.

## Results

### Aquatic community change

**Figure 2:**
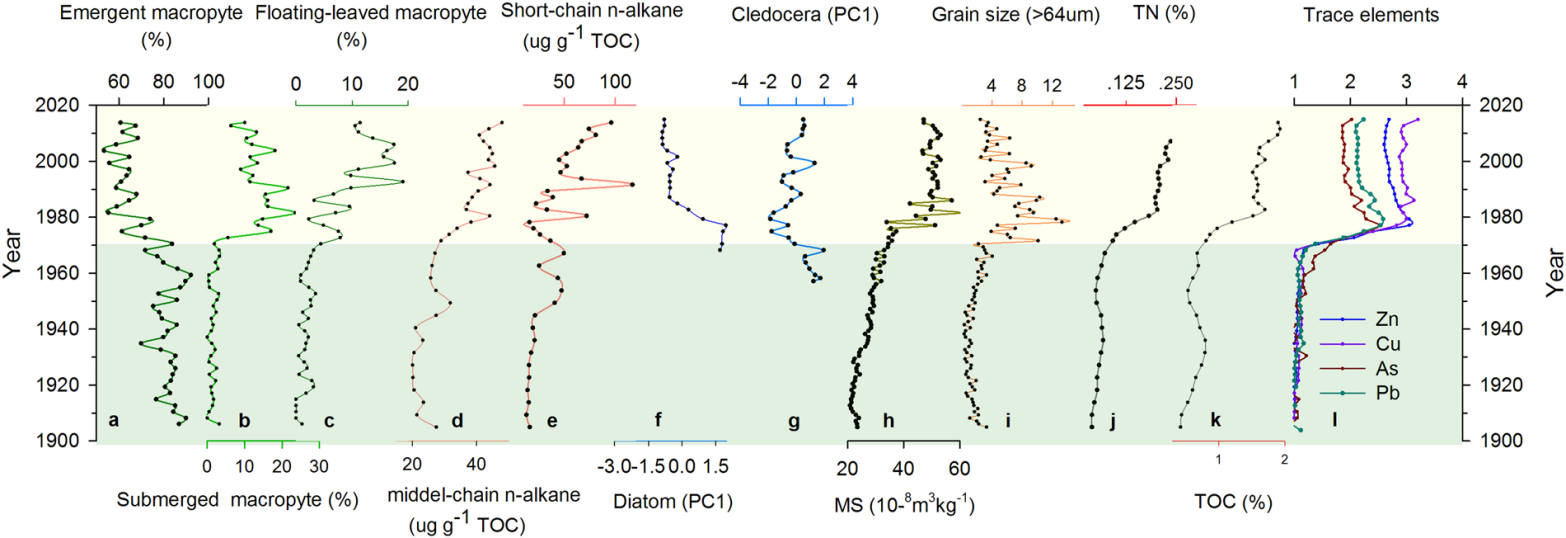
Summary of multi-proxy records from Changdang Lake. Abundance of Emergent macrophyte (**a**), Submerged macrophyte (**b**), and Floating-leaved macrophyte (**c**); Middle-chain (**d**) and short chain (**e**) of n-alkane abundance, representing aquatic macrophyte and plankton, respectively^48^; PCA1 of Diatom assemblages (**f**) and Cledocera assemblages (**g**); Magnetic susceptibility (**h**); Grain size (**i**); Total Nitrogen (**j**); Total organic carbon abundance (**k**); Enrichment factors of major trace metals (**l**).

Our results revealed substantial changes in macrophyte, zooplankton and phytoplankton communities in Changdang Lake over the last 100 years. We identified more than 20 macrophyte species (for detailed assemblage change, see Fig. S2), which can be classified into three broad groups: emergent, submerged and floating-leaved macrophyte (Fig. 2). Detailed description of aquatic pollen species change has been discussed by Ge *et al*.^34^. Cluster analysis on these pollen assemblages (Bray-Curtis distance) revealed two significant zones (Fig. S2). The relative abundances of macrophyte assemblages were quite constant and were dominant by the *Phragmites* type (with an average percentage around 60%) before the 1970s, while the submerged and floating-leaved type macrophyte increasing significantly after the 1970s.

Both the diatom and cledoceran were poorly preserved in the lower section bottom of the sediment core, only subsamples in the upper ~25 cm were counted and used for analysis in this study. There were a total of 16 Cledoceran species identified, in which Bosmina dominated the composition of the total cladocerans community up to the 1970s (Fig. S3). However, littoral species increased significantly since the 1970s, from 40% to nearly 80% around 1980s, which are dominated by *Chydorus sphaericus*. Diatom assemblages also showed clear change since 1970 (Fig. S4). The epiphytic taxa *Cocconeis placentula* (up to 60%) and the benthic *Fragilaria construnes* (up to 20%) dominated the diatom community between 1970 and 1980. Both of them declined substantially after 1980. The planktonic taxa increased significantly since 1980, characterized by *Cyclotella meneghiniana, Aulacoseira ambigua*. Facultative planktonic *Aulacoseira ambigua* reached its peak value around the 1990s.

### Environmental factors

Profiles of trace metals exhibited a broadly consistent pattern of variability (Fig. 2). Their values remained near the background levels prior to the 1970s, increased rapidly between 1970 and 1980, and subsequently decreased towards the sediment-water interface. All variables including total organic carbon (TOC), total phosphorus (TP), and total nitrogen (TN) in lake sediments were relatively stable from 1890 to 1970. However, all these variables increased abruptly between 1970 and 1980 and then kept at a relative stable level. The magnetic susceptibility (MS) and grain size of the large particle (>64 um) both showed relatively low and stable values between 1900 and 1970s and then increased abruptly during the 1970s with high variability (Fig. 2).

### Regime shift detection and pressure- response relationship

**Figure 3:**
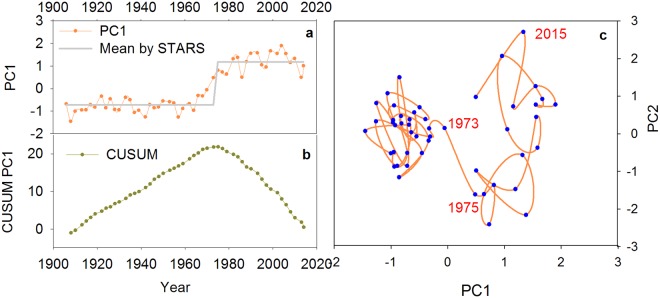
Significant ecological shifts in the macrophyte communities within the last 100 years, as revealed by STARS (**a**) and CUSUM (**b**) numerical techniques. The right panel (c) shows the phase plot of PC1 and PC2 of pollen assemblages.

**Figure 4:**
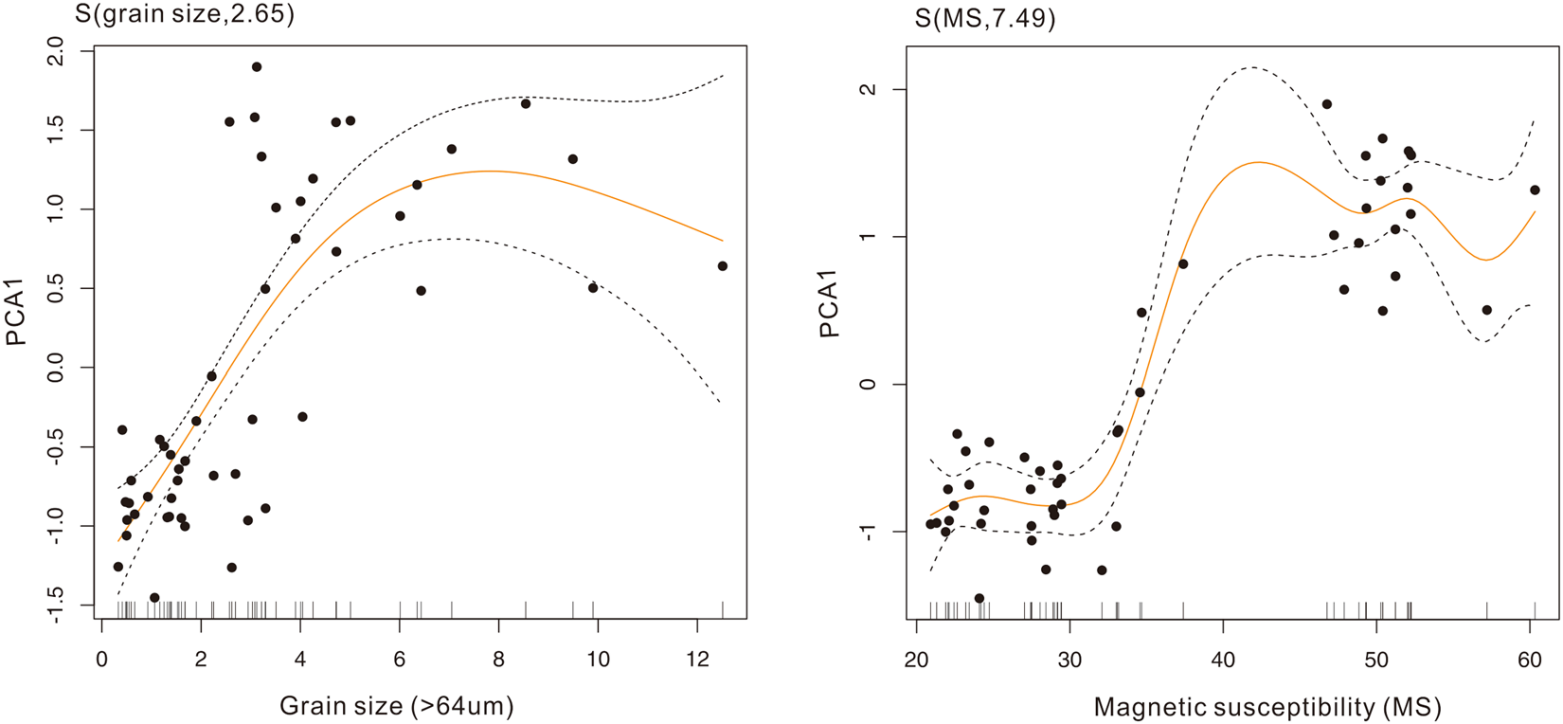
Fitted smooth function between macrophyte assemblages (PC1 scores) and grain size (left) and magnetic susceptibility (right) from a general additive model (GAM). Two dotted lines mark the 95% uncertainty interval of the fitted function. On the x-axis, ticks show the distribution of observed values for the two variables. The number in parenthesis on the y-axis (3.82, and 7.49) is the effective degrees of freedom (edf) of the smooth function.

For the aquatic macrophyte community, the first two ordination axes (PC1 and PC2) explained 58% of the variation in the aquatic pollen data set. The phase plot of the first two PCs showed two clear regimes (Fig. 3). The PC1 transition points identified by STARS were identified around the 1970s. The result from the constrained cluster analysis and CUSUM of PC1 also complemented the STARS results. The results from the STARS algorithm on PC1 of diatom and cladoceran communities also showed clear transition point between 1970 and 1980 (Fig. S5). Results from the STARS algorithms on grain size, MS, and PC1 of trace metals all identified significant breakpoint around the 1970s, though with slightly time differences (Table S1). In the generalized additive model, the aquatic assemblages (PCA1) show nonlinear relationships with grain size (F = 21.33, P < 0.001, edf = 2.65) and MS (F = 44.84, P < 0.001, edf = 7.48 corresponding to a simple sigmoid curve) (Fig. 4).

## Discussion

### Lake environment variability and abrupt ecosystem change

The analysis of multi-proxy indicators has contributed to the development of a robust reconstruction of ecosystem dynamics at the Changdang Lake over the past century. Before the 1970s, Changdang Lake was probably surrounded by an emergent macrophytic belt composed of *Typha sp*. and *Phragmites sp*. (Fig. S2) communities, while the submerged and floating-leaved macrophytic communities were relatively poor. The phytoplankton and zooplankton communities were not well developed as suggested by very low diatom and cladoceran concentrations. The lake was probably in an oligotrophic condition with low primary production. The changing frequency and magnitude of other environmental indicators (e.g. trace metals, TP, grain size, and magnetic susceptibility) also suggest relatively less anthropogenic disturbance and natural fluctuation of hydrological conditions of the lake environment. Arguably, the Changdang Lake may have stayed within its long-term historical variability prior to the 1970s (Fig. 2).

After the 1970s, the lake experienced a major transition with significant restructuring and reorganization of aquatic ecosystems, characterized by a sudden shift of the macrophyte, diatom, and cladoceran assemblages. The submerged and floating-leaved macrophytes began to expand quickly, along with an increasing abundance of benthic and epiphytic diatom species. A strong increase in the primary production was reflected by a rapid increase in the concentration of TOC and short and middle chain of n-alkane (Fig. 2). The temporal asynchrony of the abrupt change between different aquatic groups in the lake were probably due to complex biotic interactions^35^. High magnetic susceptibility suggests that increasing catchment erosion in the Changdang Lake, which may have been caused by anthropogenic activities, such as land reclamation and agricultural intensification. The increase of coarse grain size probably indicates the reduced flood energies and more stable water environment which enable large particle size from the local catchment to be deposited^36^. The sudden increase of TP and TN concentration suggest high nutrient loads, contributing to the growth of submerged macrophytes. Further, the increase in the nutrient levels many have led to the proliferation of algal and zooplankton communities. Both the submerged and emergent macrophyte declined gradually from the late 1980s, with increased planktonic diatoms species. This indicates a further degradation of the lake’s aquatic ecosystems.

The sudden shift of ecological structure, together with abrupt hydrological regime change during the 1970s, indicates a set of major environmental disturbances occurring around the lake. The results from the generalized additive model (Fig. 4) suggest that the aquatic communities variation responded to external disturbances in nonlinear ways. More than 20% of the lake area was reclaimed for agriculture land around the 1970s^37^. Widespread river regulation and construction of sluices and reservoirs within the catchment in the early 1970s substantially altered the peak flows and lake hydrology. For instance, about 20 new watercourses with dams and over 50 new water gates built along the Danjingcao River during the 1970s significantly altered the hydrologic process of Changdang Lake^37^. Stable hydrological conditions could reduce the suspension of sediments, enhancing water transparency and benefiting the development of macrophytes^38^. Similar biotic responses to reduced hydrological dynamics were also reported in the lower Yangtze regions^39,40^. Meanwhile, the increased nutrient loads from the catchment stimulated the proliferation of phytoplankton and zooplankton. Eutrophication was further amplified by industrial developments and the intensive aquaculture practices. Expansion of local industrial factories across the catchments further affected the water quality directly because of untreated sewage inputs into the lakes. Aquaculture with extensive fish cage cultures in the lake expanded to more than half of the lake area from the late 1970s^37^.

Climate change superimposed on these anthropogenic stressors and exacerbate the degraded condition further. Annual mean temperature in this region increased 1.52 °C during 1909–2010^41^. Increasing temperature has exacerbated the symptoms of eutrophication through its impact on algal productivity, water column oxygen concentrations, and nutrient recycling^42^. Overall, it is difficult to attribute any single stressor to cause the lake ecosystem shift; rather, multiple disturbances may have acted together in driving this transition.

### Dynamics of linked social-ecological systems

**Figure 5:**
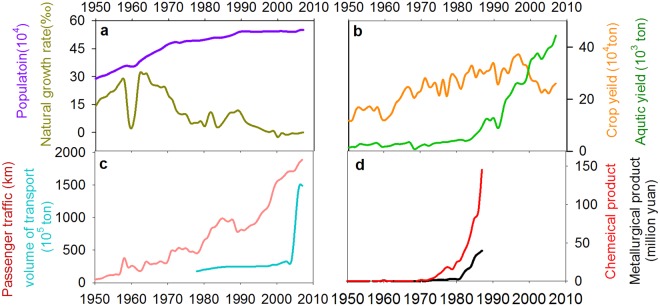
Socio-economic trends within the Changdang lake catchment system since the 1950s. Trends of at least eight socio-economic variables were examined for the lake catchment, including population and natural growth rate (**a**), crop yield and aquatic yield (**b**), passenger traffic and volume of transport (**c**), chemical product and metallurgical product (**d**).

**Figure 6:**
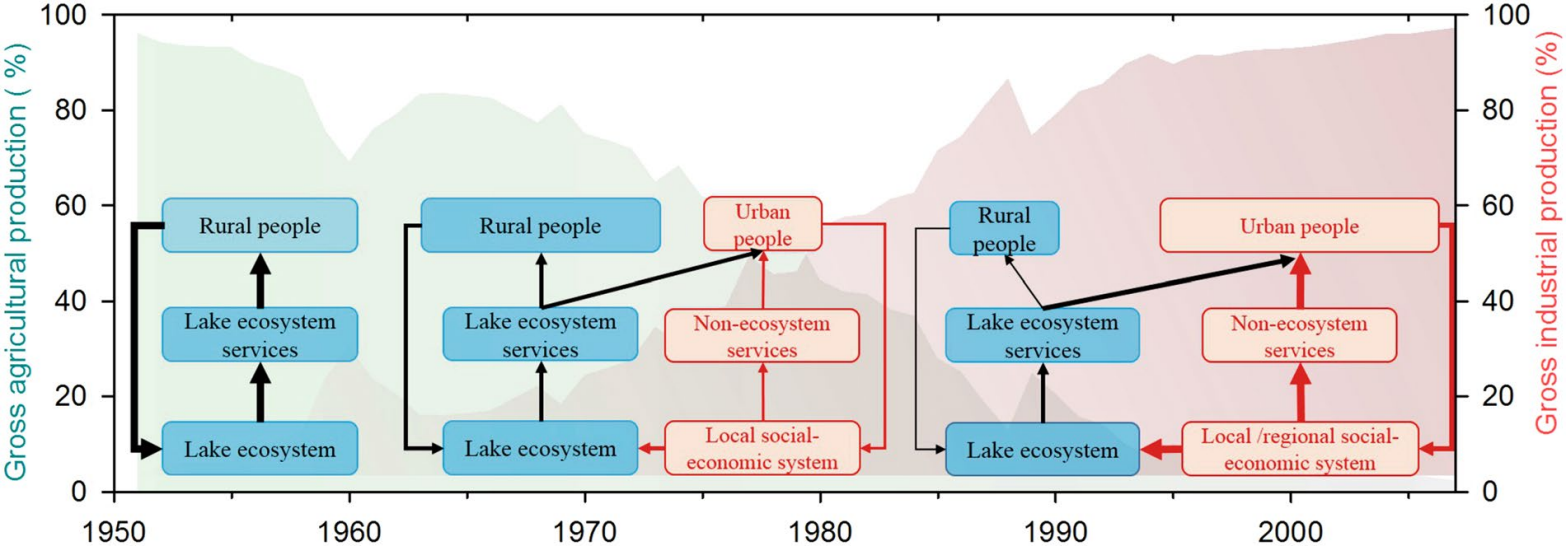
Conceptual framework shows the social-ecological transition of the Changdang Lake system with different feedback loops over the last several decades (the width of the line represent the strength of the connection, the thicker liner means a greater impact on lake ecosystems). The two Y-axes show the percentage of gross agricultural production and industrial production in this region during the past 60 years, respectively. (1) Before the 1950s, rural farmers get ecosystem services (e.g. crop, fish) directly from the local ecosystem, and the balance between local ecosystem and society was well maintained. (2) Since the 1970s, local industries began to expand rapidly, and more people work in non-agriculture sectors and become wealthy and urbanized. New relationships between local ecosystem and society were formed. Changes in socioeconomic variables, such as increased demand for food, fiber, and other resources subsequently led to a severe impact on lake ecosystems. (3) As the society became more industrialized and urbanized over the recent past (particularly within last 30 years), the strength of the two dominant feedback loops have changed. The figure is built based on Cumming^21^.

The increased nutrient loading, land reclamation, and hydrological modifications are the proximate drivers that directly triggered the shift in lake ecosystems, which is very common in many lakes within the Yangtze River Basin^29,43^. However, these proximate interactions are embedded in a much broader socioeconomic context. By integrating the historical records of societal and economic development in this region (Fig. 5), we argue that the social-ecological system went through a series of phases with complex nonlinear social and ecological interactions and feedback mechanisms (Fig. 6).

Before 1950, there was a traditional agricultural society within the Changdang lake catchment. More than 90% of the population in this region was directly employed in agricultural food production in early 1950 in Jintan County^37^. Most households relied directly on ecosystem services (fish, crops etc.) for food from local ecosystem through traditional farming and fishing^44^. Farmers produced and consumed their own food and fuels at the local scale. The interactions and feedback between lake ecosystem and human well-being via ecosystem services are relatively clear and easily understood (Fig. 6). The local equilibrium between resource use and human demand was maintained, and the anthropogenic influence on local lake ecosystem was relatively low due to low population density and limited technological development.

Population grew quickly after 1950 with increasing demand for food and resources. This caused large-scale land reclamation and hydrological modifications across the lake catchment. Meanwhile, the adoption of high-yielding technologies (fertilizer and pesticide usage, cage aquaculture) caused serious damage to the lake environment. Other non-agriculture activities, such as heavy industry expanded rapidly during this period taking many people out of agriculture-related work (Fig. 5d). The living standards have soared, and the local income has increased 11 fold during 1950–1985^37^. As a result, agriculture no longer limits the opportunities of rural households. Although the proportion of people who extracted goods directly from ecosystems (farmers, fishers) declines^37^, the resource demand increased quickly as society becomes wealthier. The consumption of fish and meat has increased 16 and 30 fold, respectively, from 1950 to 1980^37^. Local ecosystems are heavily exploited to fulfill the increasing demands of both rural and new urban people. A new feedback between society and lake ecosystem has emerged due to the rapid socio-economic change (Fig. 6).

Industrialization and urbanization developed very quickly during the past 30 years around the Changdang Lake catchment. Small villages and cities in the region have grown rapidly and merged into one of the world’s largest “megapolitan regions”. The society has transformed from an agricultural to an industrialized and urban society. Provisioning services from local ecosystems can no longer meet local demand, and many resources that were formerly met by local ecosystem are outsourced, resulting in an increase in the geographic extent of supply and demand. For instance, the transportation of goods has increased seven-fold from 1970 to 2007 in the Jintan County (Fig. 5c). Consequently, the connection between food production and food consumption has become less apparent, and society is now unintentionally placing increasing pressure on dwindling resources. The dominant feedback has changed from conventional farmer-ecosystem feedback to newly formed socio-economic and ecosystem feedback (Fig. 6). The contemporary Changdang lake ecosystem is not only impacted by local society within the catchment system but is also influenced indirectly at large regional scale through tele-coupling connections due to the market and globalization^45^.

### Management implications

Environment managers and policy-makers have a long tradition of regulating the human–environment interface with rules assuming the ecological freshwater systems, and the social systems dependent on them, are relatively linear and stable. Our analysis of the complex interactions between ecological, social, and economic systems over the past century provides several key insights for lake managers and policy-makers. First, it is impossible to understand the persistent ecosystem degradation without explicitly accounting for the interactions between the multiple dimensions of social-ecological systems at multi-decadal timescales. Though it is crucial for managers to focus on reducing direct drivers (e.g. nutrient input) to restore degraded lakes, it is also important to recognize how multiple drivers interact and create a cumulative effect, so that intervention can be targeted appropriately. Second, policy and management strategies also need to accept the likelihood that some ecosystems have changed permanently to a different configuration and fully incorporate threshold and regime shift in lake restoration and conservation^46^.

Furthermore, the long-term social-ecological trajectories and ecosystem feedback mechanisms need to be fully recognized in order to make appropriate management strategies. In the Lower Yangtze Basin, population expansion, increasing wealth and resource demands are the root causes of long-term lake ecosystem degradation. Simplistic approaches based solely on conventional natural sciences that ignore social and economic linkages are doomed to failure. For example, although the restoration of lakes to clear water conditions by reducing nutrients would is theoretically possible, the failure of considering the complex social-ecological interactions and feedbacks would create a huge social burden: the consequences for agricultural yields in the surrounding catchment may be too high to warrant the financial investment and social costs. A focus on ecological functions, ecosystem services, and human drivers, therefore, opens many more possibilities for active management intervention. Ultimately, we need to recognize that improved resource use efficiency, public environmental education, and awareness are fundamentally important for long-term sustainable lake management.

## Conclusion

In this study, we illustrate how a better understanding of the linkages between ecological and social systems at multi-decadal scales can generate critical insights for sustainable lake management. Our paleoenvironmental analysis shows abrupt shifts in the macrophyte and planktonic communities around the 1970s in the Changdang Lake. The ecological shift was corresponding to significant social and economic transition as the society transformed from traditional agricultural-base society to an industrialized and urbanized society. Rapid population and market growth, coupled with technological and policy changes, acted to intensify the direct drivers - pollution, hydrological modification and land reclamation - ultimately resulting in the shift of the Changdang lake ecosystem. These linkages - between the demands of people and dynamics of lake ecosystem - highlight the necessity of an interdisciplinary focus for effective management of lake ecosystems from a long-term perspective.

## Methods

### Study region

Changdang lake (N31°30′-31°40′, E119°30′–119°40′) is a typical shallow freshwater (mean depth 1.2 m) lake, located in the lower Yangtze River Basin, which is the most densely populated and rapidly urbanized area in China (Fig. 1). The lake area is 89 km^2^ with a drainage area of 2100 km^2^. More than 20 rivers feed into the lake and some cross the Jintan City which is 9 km away from the lake. The social and economic situation has changed dramatically in this region in the last 50 years. Population in the area has increased from 0.28 million in early 1940 to 0.55 million in 2016, and industrial output has increased from 2 million to 44 billion RMB from 1949 to 2007^37^. The rapid economic development in this region has resulted in rapid urbanization and industrialization within the Changdang lake catchment, which has had an adverse environmental impact on this lake, and others in this region^47^.

### Sample collection and Chronology

A 50 cm long sediment core was taken from the center of the Changdang Lake with a gravity corer in 2016 (Fig. 1). The core was subsampled at 0.5 cm resolution. Chronological sequences were constructed based on the radioactivities of bulk sediment ^210^Pb and ^137^Cs measured by nondestructive gamma spectrometry. ^210^Pb_ex_ generally shows an exponential distribution in the sediment core, and the increasing trend of ^210^Pb_ex_ in the upper 5 cm indicate the sediment underwent mixing or bioturbation (Fig. S1). Thus, we use the constant initial concentration model to estimate the sedimentation rate and subsequently establish an age-depth chronology. The average sedimentation rate was 0.45 cm/year and the core covers 111 years (1905–2016). Detailed chronological result has been described in Zhang *et al*.^48^.

### Pollen, Diatom and Cladoceran analysis

Pollen samples were prepared for pollen analysis following the standard acetolysis procedure of Faegri and Iversen^49^, including 10% HCl and 10% NaOH to remove carbonate and organic matter, respectively. Then they were treated by heavy liquid flotation and sieving to get rid of other impurities, acetolysis to eliminate protoplasm in pollen grains and the final preservation in glycerin. All pollen and spores were identified under light microscope with a magnification of 400X. aquatic taxa abundance was expressed with percentages.

Diatom samples preparation followed the standard procedures using the water bath technique^50^. 2 ~ 3 g freeze-dried sediment was treated with 30% H_2_O_2_ in water bathing at 85 °C, and the addition of 10% HCl to remove any carbonates. As diatoms were poorly preserved in the lower section bottom of the sediment core, only subsamples in the upper 20 cm were counted. A total of 20 samples were analyzed, and at least 300 valves were counted for each sample. Species abundance was expressed with percentages.

Cladoceran analysis followed the standard procedures^51^. Approximately 2 g dry sediment of each subsample were treated with 10% KOH solution, and heated at 60 C on a hotplate for at least 45 min. Sieving of the subsample mixture was carried out through a 38μm mesh. As Cladoceran were poorly preserved in the lower section of the sediment core, only subsamples in the upper 25 cm were counted. A total of 25 samples were analyzed, and at least 300 identifiable cladoceran remains were counted for each sample. Species abundance was expressed with percentages.

### Geochemistry analysis

Metal elements (Al, Fe, Li, K, Mg, Cr, Cu, Zn, Sr) and total phosphorus (TP) were analysed using an inductively coupled plasma-atomic emission spectrometry (ICP-AES). The CE-440 elemental analyzer (EAI Company) was used for the determination of total organic carbon (TOC) and TN content of the samples. Magnetic susceptibility (MS) was measured using a Bartington MS2C sensor. Grain size spectra of samples were determined using a Malvern automated laser optical particle-size analyzer (Mastersixer-2000). All the experiment analyses were carry out at the State Key Laboratory of Lake Science and Environment, Chinese Academy of Sciences.

### Social and economic data collection

The social and economic data of Jintan County during the last 100 year (mainly between 1949–2016) were collected from the statistic yearbook, historical document, and local county gazetteers.

### Statistical approaches

We employed multiple approaches to detecting the potential regime shifts of lake ecosystem changes. The Sequential T-test Analysis of Regime Shifts algorithm (STARS) devised by Rodionov^50,52^ was combined with classical multivariate techniques to identify statistically significant changes in the means of the paleoenvironmental temporal series. For univariate data (e.g. total organic carbon (TOC), MS), the STARS algorithm could be applied directly; while for the multivariate data (e.g. pollen, diatom, cladocero), a principal component analysis (PCA) was first used to extract major components in assemblage variation, and the algorithm was applied on the first principle component axes (PC1). Furthermore, constrained cluster analysis (*Bray-Curtis* dissimilarity) and a cumulative sum of differences (CUSUM)^53^ of these temporal variables were also run to complement with the STARS results. We also applied general additive model^54^ to investigate the relationship between aquatic macrophyte community dynamics and local anthropogenic disturbance proxies (grain size and MS).

## Electronic supplementary material

Supplementary Information

## Data Availability

The datasets generated during and/or analyzed during the current study are available from the corresponding author on reasonable request.
