# Supplementary material for: Freshwater lake ecosystem shift caused by social-economic transitions in Yangtze River Basin over the past century
Source: Sci Rep. 2018 Nov 21;8:17146. doi: 10.1038/s41598-018-35482-5 (PMC6249226; doi:10.1038/s41598-018-35482-5)

Supplementary information

Title: Freshwater lake ecosystem shift caused by social-economic transitions in Yangtze River Basin over the past century

Ke Zhang[a*], Xiangdong Yang [a], Giri Kattel [b], Qi Lin [a], Ji Shen [a]

[a] State Key Laboratory of Lake Science and Environment, Nanjing Institute of Geography and Limnology, Chinese Academy of Sciences, Nanjing 210008, China

[b] Environmental Hydrology and Water Resources Group, Department of Infrastructure Engineering, The University of Melbourne, Parkville, Victoria 3010, Australia

Table S1 Breakpoints identified in ecological and environmental variables of the Changdang Lake

Ecosystem	Breakpoint	Environmental variables	Breakpoint
Macrophyte assemblages	1973	Grain size	1970
Diatom assemblages	1979	Magnetic susceptibility	1976
Cledocera assemblages	1972	Trace metals	1970

Figure S1. The profile of the ^{210}Pb activity, age-depth model, and trace metals in the sediment core. All trace metals increased abruptly at 20 cm and then plateaued around 15cm in the sediment core. These results coincided with the initial period of industrial settings in 1970 across the catchment. Most factories were forced to shut down or reformed in 1980 due to high water pollution. This indicates the depth of 20 cm and 15 cm in the core corresponds to 1970 and 1980, respectively, and is also consistent with the ^{210}Pb results.

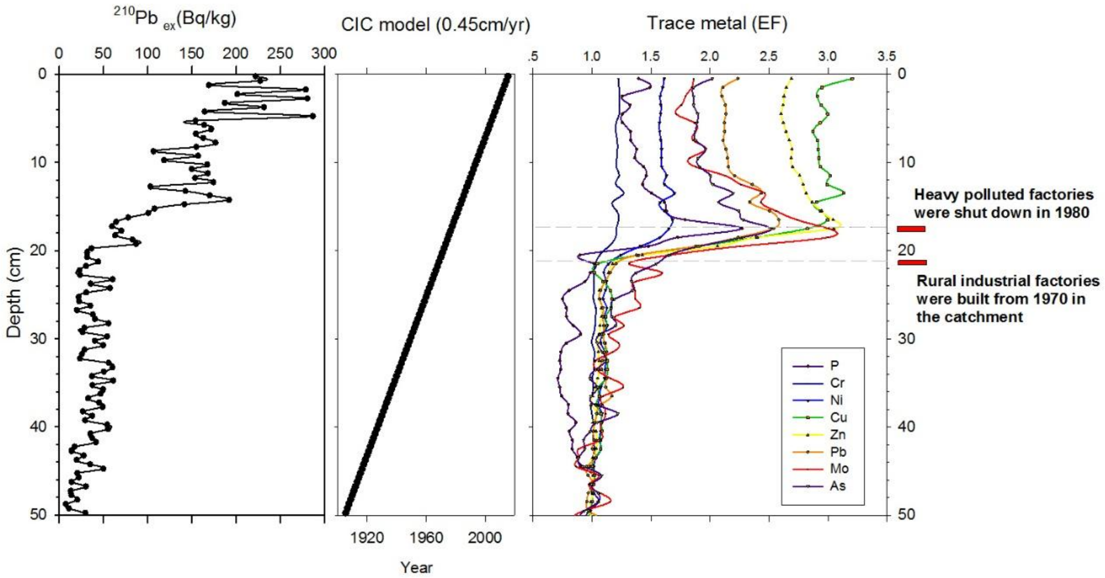

Figure S2. Summary percentage of aquatic pollen diagram of various macrophytes in Changdang Lake. Only the selected taxa have been shown in the diagram.

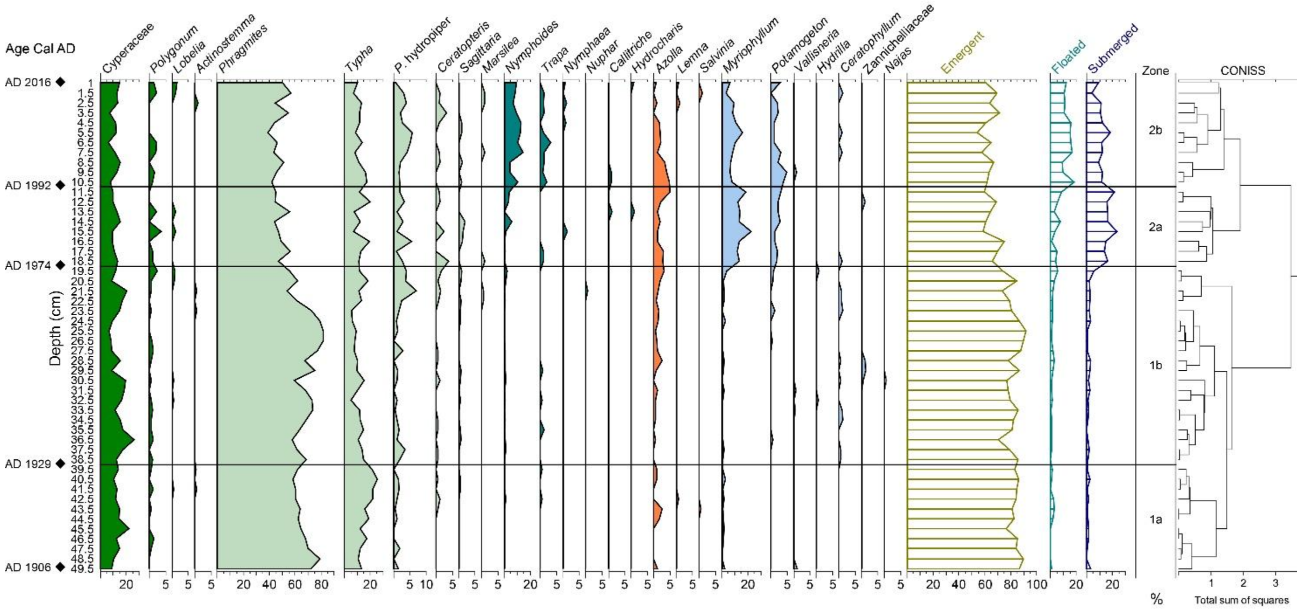

Figure S3. Summary percentage of diatom abundances in the Changdang Lake. Only selected taxa have been shown in the diagram.

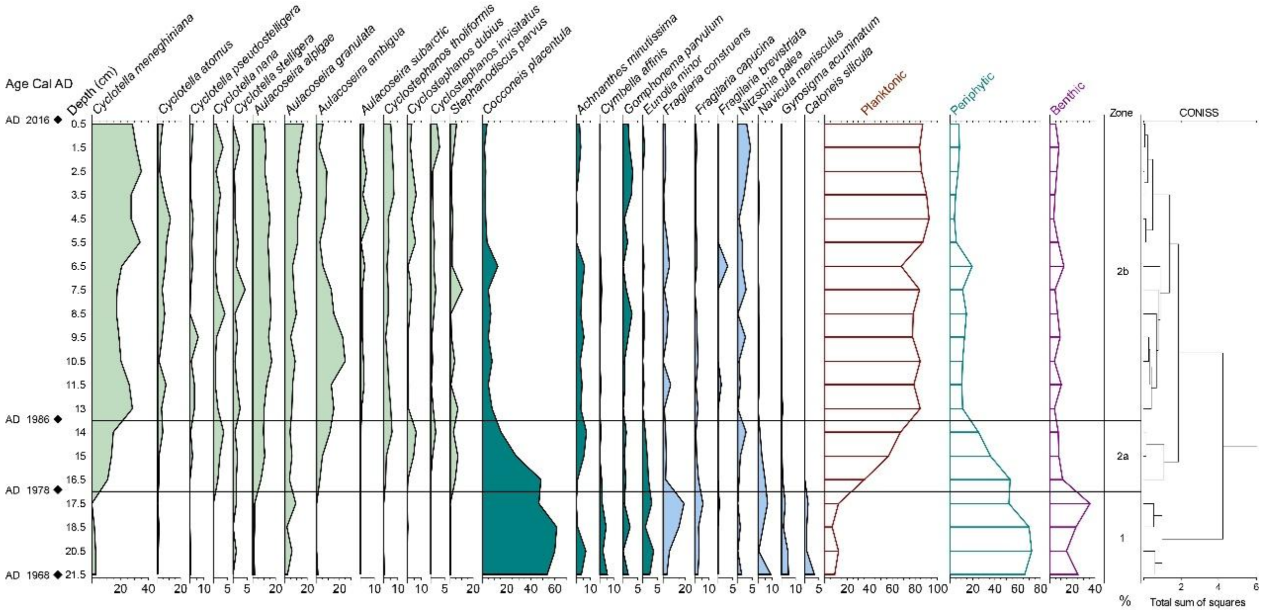

Figure S4. Summary percentage of cladoceran abundances in the Changdang Lake. Only selected taxa have been shown in the diagram.

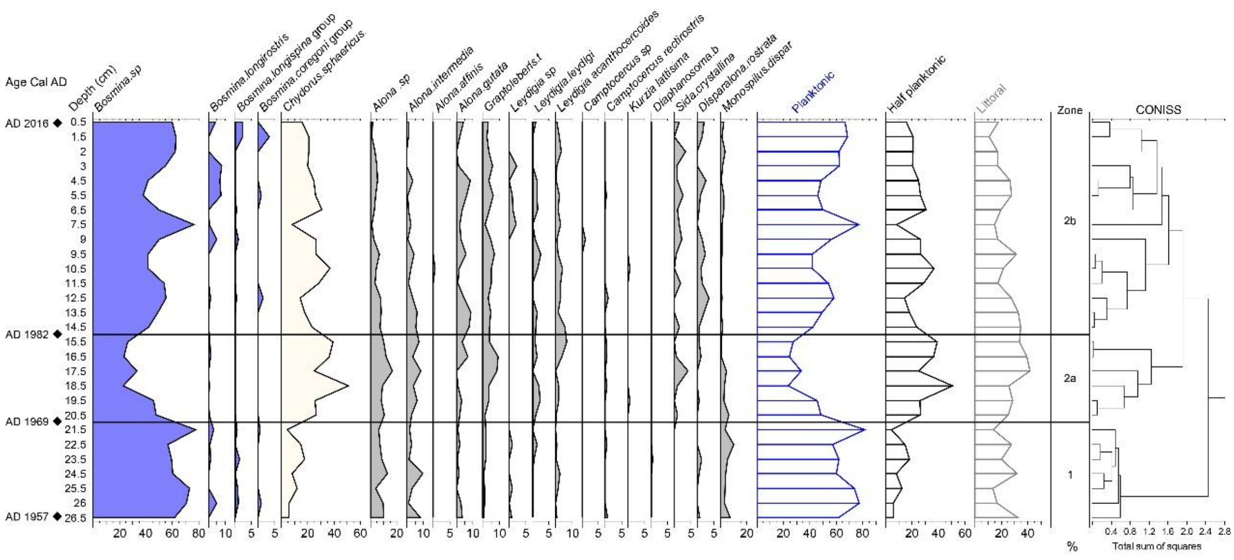

Figure S5. Significant ecological shifts in the diatom and cladocera communities within the last 100 years as revealed by STARS and CUSUM. The right panel shows the phase plot of PC1 and PC2.

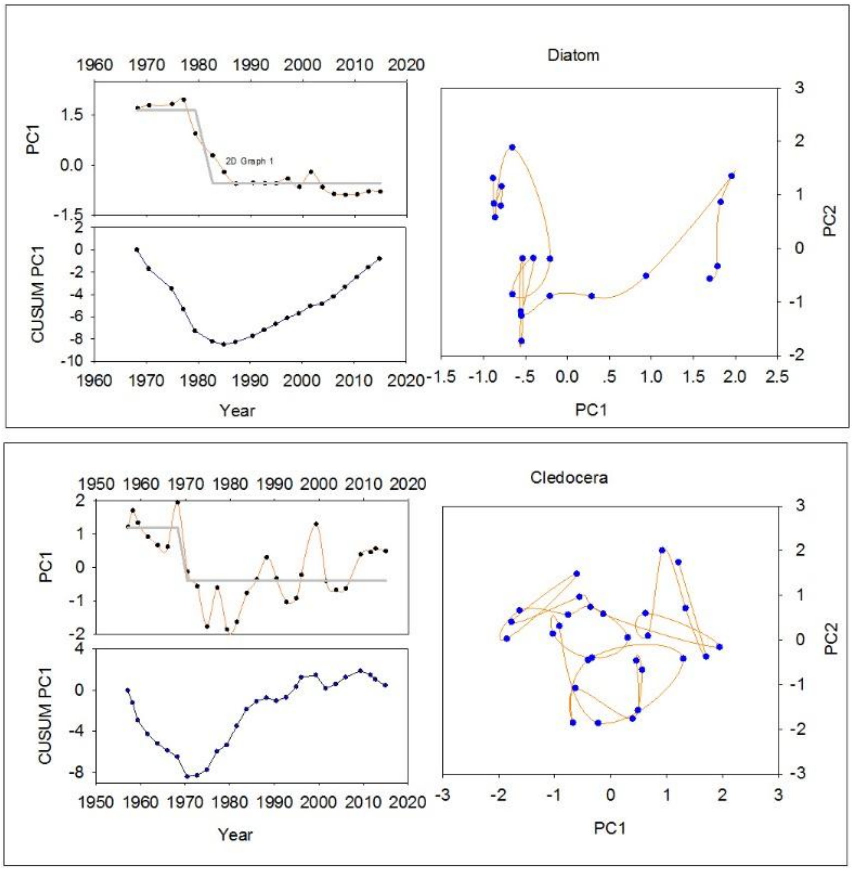

Supplement: Supplementary file 1 — Supplementary Information [file 41598_2018_35482_MOESM1_ESM.pdf]
